# Patterns of cerebellar volume loss in dementia with Lewy bodies and Alzheimer׳s disease: A VBM-DARTEL study

**DOI:** 10.1016/j.pscychresns.2014.06.006

**Published:** 2014-09-30

**Authors:** Sean. J. Colloby, John. T. O׳Brien, John-Paul Taylor

**Affiliations:** a Institute for Ageing and Health, Newcastle University, Campus for Ageing and Vitality, Newcastle upon Tyne NE4 5PL, UK; b Department of Psychiatry, University of Cambridge, Cambridge CB2 0QC, UK

**Keywords:** DARTEL-VBM, Dementia with Lewy bodies, Alzheimer’s disease, MRI, Cerebellum

## Abstract

Evidence suggests that the cerebellum contributes to cognition as well as motor function. We investigated cerebellar grey matter (GM) and white matter (WM) changes from magnetic resonance images in dementia with Lewy bodies (DLB), Alzheimer׳s disease (AD) and healthy older subjects using voxel-based morphometry (VBM). Subjects (39 controls, 41 DLB, and 48 AD) underwent magnetic resonance imaging as well as clinical and cognitive assessments. VBM used SPM8 with a cerebellar brain mask to define the subspace for voxel analysis. Statistical analyses were conducted using the general linear model. Relative to findings in controls, VBM analysis revealed cerebellar GM loss in lobule VI bilaterally in AD and in left Crus I and right Crus II regions in DLB. WM deficits were confined to AD in the bilateral middle cerebellar peduncles. DLB demonstrates a different pattern of cerebellar GM loss which, although not significantly different from that in AD, could be an important feature in understanding the neurobiology of DLB and warrants further investigation.

## Highlights

• We studied cerebellar grey and white matter changes from MR images in dementia.
• Cerebellar volumes loss occurred in both DLB and AD compared to controls.
• Cerebellar pathology under both conditions warrants further consideration.

## 1 Introduction

To date, voxel based morphometry (VBM) studies in patients with dementia with Lewy bodies (DLB) when contrasted against patients with Alzheimer׳s disease (AD) have focussed on volumetric changes within the cerebrum. Patterns of cortical atrophy in AD are typically in the medial temporal lobe and temporoparietal association cortices (Burton et al., 2002, Karas et al., 2003, Whitwell et al., 2007, Takahashi et al., 2010, Watson et al., 2012). In DLB, there is some overlap with the AD pattern, but atrophy is less diffuse, with relative preservation of the medial temporal lobe (Burton et al., 2002, Karas et al., 2003, Whitwell et al., 2007, Takahashi et al., 2010, Watson et al., 2012), and there is also evidence of greater subcortical atrophy (Cousins et al., 2003, Hanyu et al., 2007, Whitwell et al., 2007).

What is less established, however, is whether there are volumetric changes in the cerebellum in DLB. This is an important question as it is now recognised that the cerebellum has a significant role beyond sensorimotor control in cognition and affect (Schmahmann and Caplan, 2006). For example, there is evidence that the cerebellum, by means of extensive cerebellar-cortical connections, participates in distributed cognitive networks which include structures such as the prefrontal and parietal association cortices (Stoodley, 2012). Functionally, these networks may impact executive functioning and predictive motor timing, both of which may have important implications for conditions such as Parkinson׳s disease and, by extension, DLB (Husarova et al., 2013, Nombela et al., 2013).

Pathologically, cerebellar atrophy has been observed in other related alpha-synuclein pathologies such as Parkinson׳s disease (Pereira et al., 2009), multi-system atrophy with cerebellar features and idiopathic rapid eye movement (REM) sleep behaviour disorder (iRBD) (Schulz et al., 1994). Cerebellar volumetric and grey matter (GM) density reductions have been observed in progressive supranuclear palsy (Agosta et al., 2010, Messina et al., 2011, Giordano et al., 2013) as well as conditions such as schizophrenia (Chua et al., 2007, Kuhn et al., 2012), thus further highlighting the potential importance of the cerebellum in diseases with significant cognitive, motor and neuropsychiatric sequelae. In DLB, cerebellar alpha-synuclein deposition affecting both purkinje and glial cells has been reported (Mori et al., 2003), and more recently, functional metabolic and perfusion imaging changes have suggested that cerebellar uptake along with uptake in other motor areas may be associated with cognitive and attentional fluctuations (Taylor et al., 2013) as well as visual hallucinations (Miyazawa et al., 2010).

Volumetric cerebellar changes in DLB, however, have not been systematically reported. Therefore, in the present study we performed separate GM and white matter (WM) assessment of the cerebellum in DLB, AD and healthy older subjects as well as their clinical correlates using diffeomorphic anatomical registration with exponentiated Lie algebra (DARTEL) voxel-based morphometry (VBM).

## 2 Methods

### 2.1 Participants

Eighty-eight individuals over the age of 60 (47 subjects with probable AD (McKhann et al., 1984), 41 with probable DLB (McKeith et al., 2005)) were recruited from a community dwelling population of patients referred to local Old Age Psychiatry, Geriatric Medicine or Neurology Services. All subjects underwent clinical and neuropsychological assessments. Thirty-nine healthy controls of similar ages were recruited from among relatives and friends of patients with dementia. The research was approved by the local ethics committee. All subjects or, where appropriate, their nearest relative provided written informed consent. Exclusion criteria for all subjects included contra-indications for magnetic resonance imaging (MRI), previous history of alcohol or substance misuse, significant neurological or psychiatric history, focal brain lesions on brain imaging, or the presence of other severe or unstable medical illness.

Assessment of global cognitive measures included the Cambridge Cognitive Examination (CAMCOG), incorporating the Mini-Mental State Examination (MMSE) (Folstein et al., 1975). Motor parkinsonism was measured with the Unified Parkinson׳s Disease Rating Scale Part III (UPDRS-III) (Fahn and Elton, 1987). For participants with dementia, neuropsychiatric features were assessed using the Neuropsychiatric Inventory (NPI) (Cummings et al., 1994), while cognitive fluctuations were examined using the clinician׳s assessment of fluctuation (Walker et al., 2000).

### 2.2 MRI data acquisition

All subjects underwent T1-weighted MR scanning on a 3-Tesla MRI system using an eight-channel head coil (Intera Achieva scanner, Philips Medical Systems, Eindhoven, Netherlands). The dataset was generated from two independent imaging cohorts (cohort 1: controls 23, AD 31, DLB 23; cohort 2: controls 16, AD 16, DLB 18) with slightly different T1 sequences. The sequence for cohort 1 was as follows: whole brain, 3D MPRAGE, sagittal acquisition, matrix size 216 (anterior–posterior)×208 (superior–inferior)×180 (right–left), repetition time (TR)=8.3 ms, echo time (TE)=4.6 ms, inversion time (TI)=1250 ms, flip angle=8°, SENSE factor=2, voxel output 1×1×1 mm^3^. The sequence for cohort 2 was as follows: whole brain, 3D MPRAGE, sagittal acquisition, matrix size 240 (anterior–posterior)×240 (superior–inferior)×150 (right–left), TR=9.6 ms, TE=4.6 ms, TI=1250 ms, flip angle=8°, SENSE factor=2, voxel output 0.94×0.94×1.2 mm^3^.

### 2.3 VBM-DARTEL analysis

VBM analysis was conducted using SPM8 (http://www.fil.ion.ucl.ac.uk/spm) running on MATLAB 7.9 (Math-Works, Natick, MA, USA). First, MR images were segmented into GM, WM and cerebrospinal fluid (CSF) using SPM8׳s standard unified segmentation module (Ashburner and Friston, 2005). Second, population templates (GM, WM) were derived from the entire image dataset using the DARTEL technique (Ashburner, 2007). Third, after an initial affine registration of the DARTEL templates to the corresponding tissue probability maps in Montreal Neurological Institute (MNI) space (http://www.mni.mcgill.ca/), non-linear warping of the segmented images was then performed to match the corresponding MNI space DARTEL templates (GM, WM). Fourth, images were modulated to ensure that relative volumes of GM and WM were preserved following spatial normalisation. Lastly, images were smoothed with an 8-mm full width at half maximum Gaussian kernel. After spatial pre-processing, the smoothed, modulated, normalised imaging datasets were then used for voxelwise statistical analysis.

### 2.4 Statistical analysis

Group differences in GM and WM volumes were assessed using the general linear model in SPM8, and statistical significance was estimated from the distributional approximations of Gaussian random fields (Friston et al., 1994). Age and total intracranial volume (TIV_SPM8_) were entered into the design matrix as nuisance variables. Multiple regression analyses were also performed to investigate effects of GM and WM loss on clinical and cognitive variables separately in AD and DLB. A cerebellum binary mask image obtained from the Wake Forest University Pickatlas toolbox (http://fmri.wfubmc.edu/software/PickAtlas) defined the brain volume subspace for all voxel analyses. Significant effects were identified using an uncorrected threshold (*P*_uncorrected_≤0.001), followed by correction for multiple comparisons using the family-wise error (FWE) (*P*_FWE_≤0.05) within the cerebellar volume subspace.

For demographic and clinical data, the Statistical Package for the Social Sciences software (SPSS ver. 19.0.0.1, http://www-01.ibm.com/software/analytics/spss/) was used for further statistical evaluation. Continuous variables were tested for normality using the Shapiro–Wilk test and visual inspection of variable histograms, and assessed where appropriate using parametric (analysis of variance, ANOVA) and non-parametric (Kruskal–Wallis) procedures. For categorical data, χ^2^ tests were applied.

## 3 Results

### 3.1 Subject characteristics

Table 1 shows demographic and group characteristics. Groups were matched for age and gender. CAMCOG and MMSE scores were similar between AD and DLB patients but significantly differed from scores of controls. As expected, UPDRS III measures were significantly higher in DLB patients than in AD patients and controls. NPI, NPI_hallucinations, CAF scores and frequency of RBD were all significantly greater in DLB than AD. The proportion of individuals receiving cholinesterase inhibitors did not significantly differ between dementia groups.

**Table 1:** Demographic and group characteristics.

	Control	AD	DLB	Statistic, *P* value
*n*	39	47	41	
Gender (m: f)	25: 14	33: 14	26: 15	χ^2^=0.6, 0.8
Age (years)	77.0±6.4	79.0±8.8	78.6±6.2	*F*_2,124_=0.8, 0.4
MMSE	29.0±1.0	20.8±4.0	20.9±5.0	***F***_**2,124**_**=62.0,** <**0.001**†
CAMCOG	96.5±3.3	67.8±13.5	69.5±14.9	***F***_**2,124**_**=74.1,** <**0.001**†
NPI total	Na	9.3±8.7	13.5±11.0	***U***_**88**_**=1026.5, 0.04**
NPI_hall	Na	0.2±0.8	2.1±2.1	***U***_**88**_**=1521.0,** <**0.001**
RBD (y: n)	0: 39	1: 46	23: 18	**χ**^**2**^**=22.6,** <**0.001**⁎
UPDRS III	1.2±1.7	2.6±2.4	24.4±13.7	***H***_**2**_**=86.1,** <**0.001**††
CAF	Na	1.5±3.0	5.9±4.7	***U***_**88**_**=1380.5,** <**0.001**
ChI use (y: n)	Na	40: 7	32: 9	χ^2^=0.7, 0.4
TIV_spm8_ (ml)	1500.0±133.8	1495.4±134.0	1525.0±154.4	*F*_2,124_=0.4, 0.6

**Bold** text denotes statistical significance.

Values expressed as Mean±1 S.D.

MMSE=Mini Mental State Examination, CAMCOG=Cambridge Cognitive Examination, NPI=Neuropsychiatric Inventory, NPI_hall=NPI hallucinations, RBD=REM sleep behaviour disorder, UPDRS III=Unified Parkinson׳s Disease Rating Scale (section III), ChI=cholinesterase inhibitor, TIV=total intracranial volume. CAF=Clinical Assessment of Fluctuation. Na=not applicable.

Post-hoc tests:

⁎ AD vs. DLB.

† Con>AD, DLB (*P*<0.001), AD vs. DLB (*P*>0.10) (Gabriel׳s).

†† DLB>Con, AD (*P*<0.001), Con vs. AD (*P*=0.14) (Mann–Whitney *U*).

### 3.2 Cerebellar GM and WM analysis

**Fig. 1:**
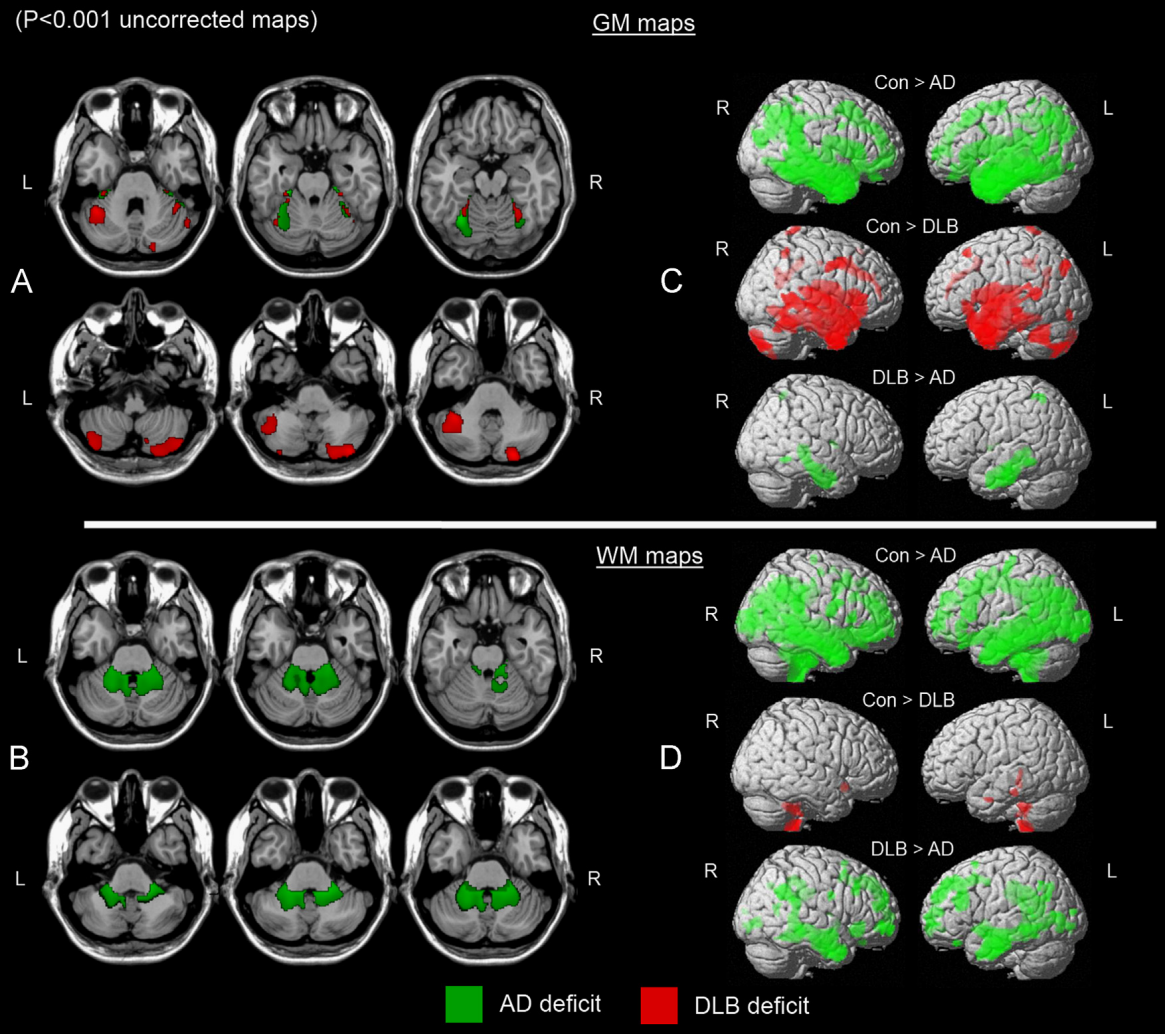
Significant cerebellar GM loss in AD (green) and DLB (red) relative to healthy older subjects (A). Significant cerebellar WM loss in AD relative to healthy older subjects (B). Whole brain maps depicting significant regions of GM (C) and WM (D) loss between groups. Results superimposed on a MRI T1 brain template image (L=left, R=right).(For interpretation of the references to colour in this figure legend,the reader is referred to the web version of this article)

Relative to findings in controls, SPM8 revealed significant cerebellar GM loss in AD in lobule VI bilaterally, extending into parahippocampal and fusiform structures (Fig. 1A, green map, *P*_FWE_≤0.05). In DLB, a different pattern of GM loss emerged compared with controls in the left Crus I and right Crus II regions, extending to a lesser extent into fusiform structures (Fig. 1A, red map, *P*_FWE_≤0.05). Significant differences were not observed, however, between AD and DLB for either contrast (AD>DLB, DLB>AD), and cerebellar GM atrophy in controls did not exceed that of AD or DLB. In WM, deficits were observed in AD participants in the bilateral middle cerebellar peduncles and the left inferior cerebellar peduncle (Fig. 1B, green map, *P*_FWE_≤0.05). No WM deficits were identified in controls that were greater than those in AD. WM loss did not significantly differ between DLB and controls or among dementia groups for any contrasts (Con>DLB, DLB>Con, AD>DLB, DLB>AD). Table 2 shows the location and peak significance of statistical maps across selected groups. There were no significant associations between any clinical and cognitive measures (CAMCOG, MMSE, NPI, UPDRS III and CAF scores) and GM volume in AD or DLB. Effects of WM volume loss on these variables were also examined in AD and DLB, yielding non-significant results.

**Table 2:** Location and peak significance of cerebellar GM and WM volume loss in selected groups using VBM-DARTEL.

	Voxel-level (*P*_FWE-corr_)	Extent (*k*)	*t, Z*	MNI coordinates (*x*,*y*,*z*) (mm)	Region
	***GM***				
Controls vs. AD	<0.001	1330	7.3, 6.4	−29,–30, −24	Left parahippocampus
	0.02		4.6, 4.3	−33, −43, −24	Left VI
	<0.001	739	6.2, 5.6	29, −31, −24	Right fusiform
	0.02		4.5, 4.3	42, −43, −26	Right VI
Controls vs. DLB	0.01	1672	4.7, 4.4	21, −85, −35	Right Crus II
	0.03		4.5, 4.2	39, −79, −44	Right Crus II
	0.02	1965	4.6, 4.3	−41, −48, −32	Left Crus I
	0.02	205	4.6, 4.3	−27, −37, −20	Left fusiform
	0.03	447	4.4, 4.1	26, −37, −20	Right fusiform
AD vs. DLB	No significant differences				
	***WM***				
Controls vs. AD	0.003	5195	5.0, 4.7	21, −36, −39	RM cerebellar peduncle
	0.004		4.9, 4.6	17, −42, −36	RM cerebellar peduncle
	0.008		4.7, 4.4	−20, −40, −35	LM cerebellar peduncle
	0.01		4.6, 4.3	−9, −42, −35	LI cerebellar peduncle
	0.01		4.6, 4.3	18, −39, −32	RM cerebellar peduncle
Controls vs. DLB	No significant differences				
AD vs. DLB	No significant differences				

*Note:* Table depicts voxel-level significance (*P*_FWE-corr_), spatial extent (*k*), *t* and *Z* scores, MNI coordinates and anatomical region. RM=right middle, LM=left middle and LI=left inferior.

To characterise the cerebellar results, whole-brain difference maps between groups depicting AD loss compared with controls (Con>AD), DLB loss relative to controls (Con>DLB) and AD loss compared with DLB (DLB>AD) are also presented for GM (Fig. 1C) and WM (Fig. 1D).

## 4 Discussion

To our knowledge, this was the first study to investigate and observe patterns of cerebellar GM and WM atrophy in DLB and AD compared with healthy subjects of similar age using DARTEL-VBM. Two results emerged from this analysis. First, DLB had a pattern of cerebellar GM loss distinct but not significantly different from AD. Second, compared with findings in controls, WM cerebellar deficits were apparent in AD but not in DLB. GM matter loss has been previously reported across the continuum from mild cognitive impairment to AD (Thomann et al., 2008, Dos Santos et al., 2011, Spulber et al., 2012, Moller et al., 2013), and thus our findings are consistent with these. Since total GM volumes are proportional to total WM volumes in both AD and DLB (AD: *r*=0.55, *P*<0.001; DLB: *r*=0.63, *P*<0.001), WM loss in AD was not unexpected given the loss of cortico-cerebellar connections that are likely to occur as a result of the significant cortical GM loss under this condition which could impact upon the widespread connectivity between the cortex and cerebellum. By similar argument, there was less cortical atrophy in DLB, and this may potentially explain the lack of cerebellar WM loss observed in DLB compared with controls. The pattern of GM loss in DLB compared with the pattern in controls primarily involved the posterior and lateral cerebellum, areas which have been associated with cognitive functions such as executive function and working memory (Stoodley et al., 2010), and atrophy in these areas has also been implicated in psychopathology and thought disorder observed in schizophrenia (Schmahmann, 1991, Andreasen et al., 1998) as well as with cerebellar ‘dysmetria of thought’ (Schmahmann, 1991); the latter is a concept that implies that in addition to the canonical motor function of the cerebellum to regulate force, rhythm, timing and accuracy of movement, the cerebellum also performs an analogous regulatory role for higher cognitive and mental functions (Schmahmann, 1998).

In the present study, we did not observe any associations between the severity of volumetric change (GM or WM) and a wide range of clinical variables including cognitive and neuropsychiatric measures in either DLB or AD. It could be that the observed cerebellar structural changes are epiphenomena which are not central to the clinical phenotypes seen in either disease state or that cerebellar atrophy, in itself, is not necessarily relevant to cognitive and neuropsychiatric symptoms in DLB/AD, but more a representation of the broader neurodegenerative disease process. However, alterations in functional state of the cerebellum rather than structure may be more salient to cognitive and psychiatric symptomatology in DLB; for example, there is a reported association between cerebellar hypermetabolism in DLB and visual hallucinations (Miyazawa et al., 2010). Alternatively, it may be that the role of the cerebellum should be considered as only one element in a distributed cognitive-motor network given the major inter-connection between the cerebellum and the cerebrum (Miyazawa et al., 2010); thus, multivariate network approaches may be more sensitive in elucidating any cerebellar contribution to the cognitive/neuropsychiatric phenotypes of DLB (Taylor et al., 2013). Finally, it may also be that the global clinical assessments applied in the present study (e.g., CAMCOG and MMSE) are not specific to those cognitive or neuropsychiatric features which may depend upon cerebellar function; for example, there may be a need for specific tests that tune into cognitive domains known to be sensitive to cerebellar dysfunction such as working memory tasks (Tomlinson et al., 2014). Similarly the application of more detailed neuropsychological test batteries that are sensitive and specific to the differing cognitive profiles of AD and DLB may help delineate what functions may map onto specific patterns of cerebellar atrophy.

In comparison of the two forms of dementia, we observed no significant differential patterns of cerebellar atrophy between AD and DLB despite different patterns of atrophy compared with controls. The absence of a differential pattern for each disorder is unlikely to have been driven by type II errors given our relatively large patient samples; rather, it could reflect the heterogeneity in the degree of cerebellar loss seen in these dementias and commonalities between AD and DLB in terms of neurogenerative processes such as amyloid deposition (Quigley et al., 2011). Our findings of volumetric changes in the cerebellum may also have implications for functional imaging studies in dementia, where this structure has been used as a reference region for image-intensity normalisation (Lacalle-Aurioles et al., 2013).

Strengths of the current study include the following: relatively large AD and DLB cohorts, 3-Tesla MRI dataset, and rigorous and validated methodologies for imaging. Patients were diagnostically classified by two independent raters, and underwent detailed clinical and cognitive assessment. The data also comprised two slightly different T1 sequences, so in addition to age and TIV, sequence was also included as a nuisance covariate; however, this did not affect results and was therefore excluded from the model. One potential weakness may lie in the inter-subject cerebellar spatial normalisation that was implemented by the whole-brain DARTEL algorithm rather than a cerebellum-optimised approach (Diedrichsen, 2006). The optimised procedure uses spatially unbiased templates that could offer increased sensitivity, though this did not appear to be a limiting factor in the present study. In conclusion, we observed clear volumetric cerebellar losses in both DLB and AD compared with controls, suggesting that this structure is affected by the neurogenerative processes in both disease states. The potential impact of cerebellar pathology in the broader clinical phenotype of the two conditions warrants further consideration.

## References

[bib1] Agosta F., Kostic V.S., Galantucci S., Mesaros S., Svetel M., Pagani E., Stefanova E., Filippi M. (2010). The in vivo distribution of brain tissue loss in Richardson׳s syndrome and PSP-parkinsonism: a VBM-DARTEL study. The European Journal of Neuroscience.

[bib2] Andreasen N.C., Paradiso S., O׳Leary D.S. (1998). Cognitive dysmetria” as an integrative theory of schizophrenia: a dysfunction in cortical-subcortical-cerebellar circuitry. Schizophrenia Bulletin.

[bib3] Ashburner J. (2007). A fast diffeomorphic image registration algorithm. Neuroimage.

[bib4] Ashburner J., Friston K.J. (2005). Unified segmentation. Neuroimage.

[bib5] Burton E.J., Karas G., Paling S.M., Barber R., Williams E.D., Ballard C.G., McKeith I.G., Scheltens P., Barkhof F., O׳Brien J.T. (2002). Patterns of cerebral atrophy in dementia with Lewy bodies using voxel-based morphometry. Neuroimage.

[bib6] Chua S.E., Cheung C., Cheung V., Tsang J.T., Chen E.Y., Wong J.C., Cheung J.P., Yip L., Tai K.S., Suckling J., McAlonan G.M. (2007). Cerebral grey, white matter and csf in never-medicated, first-episode schizophrenia. Schizophrenia Research.

[bib7] Cousins D.A., Burton E.J., Burn D., Gholkar A., McKeith I.G., O׳Brien J.T. (2003). Atrophy of the putamen in dementia with Lewy bodies but not Alzheimer׳s disease: an MRI study. Neurology.

[bib8] Cummings J.L., Mega M., Gray K., Rosenberg-Thompson S., Carusi D.A., Gornbein J. (1994). The Neuropsychiatric Inventory: comprehensive assessment of psychopathology in dementia. Neurology.

[bib9] Diedrichsen J. (2006). A spatially unbiased atlas template of the human cerebellum. Neuroimage.

[bib10] Dos Santos V., Thomann P.A., Wustenberg T., Seidl U., Essig M., Schroder J. (2011). Morphological cerebral correlates of CERAD test performance in mild cognitive impairment and Alzheimer׳s disease. Journal of Alzheimer׳s Disease.

[bib11] Fahn S., Elton R., Fahn S., Marsden C., Calne D., Goldstein M. (1987). Recent Developments in Parkinson׳s Disease.

[bib12] Folstein M., Folstein S., McHugh P. (1975). “Mini-mental state”. A practical method for grading the cognitive state of patients for the clinician. Journal of Psychiatric Research.

[bib13] Friston K.J., Worsley K.J., Frackowiak R.S.J., Mazziotta J.C., Evans A.C. (1994). Assessing the significance of focal activations using their spatial extent. Human Brain Mapping.

[bib14] Giordano A., Tessitore A., Corbo D., Cirillo G., de Micco R., Russo A., Liguori S., Cirillo M., Esposito F., Tedeschi G. (2013). Clinical and cognitive correlations of regional gray matter atrophy in progressive supranuclear palsy. Parkinsonism & Related Disorders.

[bib15] Hanyu H., Shimizu S., Tanaka Y., Hirao K., Iwamoto T., Abe K. (2007). MR features of the substantia innominata and therapeutic implications in dementias. Neurobiology of Aging.

[bib16] Husarova I., Mikl M., Lungu O.V., Marecek R., Vanicek J., Bares M. (2013). Similar circuits but different connectivity patterns between the cerebellum, basal ganglia, and supplementary motor area in early Parkinson׳s disease patients and controls during predictive motor timing. Journal of Neuroimaging.

[bib17] Karas G.B., Burton E.J., Rombouts S., van Schijndel R.A., O׳Brien J.T., Scheltens P., McKeith I.G., Williams D., Ballard C., Barkhof F. (2003). A comprehensive study of gray matter loss in patients with Alzheimer׳s disease using optimized voxel-based morphometry. Neuroimage.

[bib18] Kuhn S., Romanowski A., Schubert F., Gallinat J. (2012). Reduction of cerebellar grey matter in Crus I and II in schizophrenia. Brain Structure & Function.

[bib19] Lacalle-Aurioles M., Aleman-Gomez Y., Guzman-De-Villoria J.A., Cruz-Orduna I., Olazaran J., Mateos-Perez J.M., Martino M.E., Desco M. (2013). Is the cerebellum the optimal reference region for intensity normalization of perfusion MR studies in early Alzheimer׳s Disease?. PLoS One.

[bib20] McKeith I.G., Dickson D.W., Lowe J., Emre M., O׳Brien J.T., Feldman H., Cummings J., Duda J.E., Lippa C., Perry E.K., Aarsland D., Arai H., Ballard C.G., Boeve B., Burn D.J., Costa D., Del Ser T., Dubois B., Galasko D., Gauthier S., Goetz C.G., Gomez-Tortosa E., Halliday G., Hansen L.A., Hardy J., Iwatsubo T., Kalaria R.N., Kaufer D., Kenny R.A., Korczyn A., Kosaka K., Lee V.M., Lees A., Litvan I., Londos E., Lopez O.L., Minoshima S., Mizuno Y., Molina J.A., Mukaetova-Ladinska E.B., Pasquier F., Perry R.H., Schulz J.B., Trojanowski J.Q., Yamada M. (2005). Diagnosis and management of dementia with Lewy bodies: third report of the DLB consortium. Neurology.

[bib21] McKhann G., Drachman D., Folstein M., Katzman R., Price D., Stadlan E. (1984). Clinical diagnosis of Alzheimer׳s disease: report of the NINCDS-ADRDA work group under the auspices of department of health and human services task force on Alzheimer׳s disease. Neurology.

[bib22] Messina D., Cerasa A., Condino F., Arabia G., Novellino F., Nicoletti G., Salsone M., Morelli M., Lanza P.L., Quattrone A. (2011). Patterns of brain atrophy in Parkinson׳s disease, progressive supranuclear palsy and multiple system atrophy. Parkinsonism & Related Disorders.

[bib23] Miyazawa N., Shinohara T., Nagasaka T., Hayashi M. (2010). Hypermetabolism in patients with dementia with Lewy bodies. Clinical Nuclear Medicine.

[bib24] Moller C., Vrenken H., Jiskoot L., Versteeg A., Barkhof F., Scheltens P., van der Flier W.M. (2013). Different patterns of gray matter atrophy in early- and late-onset Alzheimer׳s disease. Neurobiology of Aging.

[bib25] Mori F., Piao Y.S., Hayashi S., Fujiwara H., Hasegawa M., Yoshimoto M., Iwatsubo T., Takahashi H., Wakabayashi K. (2003). Alpha-synuclein accumulates in Purkinje cells in Lewy body disease but not in multiple system atrophy. Journal of Neuropathology and Experimental Neurology.

[bib26] Nombela C., Hughes L.E., Owen A.M., Grahn J.A. (2013). Into the groove: can rhythm influence Parkinson׳s disease?. Neuroscience and biobehavioral reviews.

[bib27] Pereira J.B., Junque C., Marti M.J., Ramirez-Ruiz B., Bartres-Faz D., Tolosa E. (2009). Structural brain correlates of verbal fluency in Parkinson׳s disease. Neuroreport.

[bib28] Quigley H., Colloby S.J., O׳Brien J.T. (2011). PET imaging of brain amyloid in dementia: a review. International Journal of Geriatric Psychiatry.

[bib29] Schmahmann J.D. (1991). An emerging concept. The cerebellar contribution to higher function. Archives of Neurology.

[bib30] Schmahmann J.D. (1998). Dysmetria of thought: clinical consequences of cerebellar dysfunction on cognition and affect. Trends in Cognitive Sciences.

[bib31] Schmahmann J.D., Caplan D. (2006). Cognition, emotion and the cerebellum. Brain.

[bib32] Schulz J.B., Klockgether T., Petersen D., Jauch M., Muller-Schauenburg W., Spieker S., Voigt K., Dichgans J. (1994). Multiple system atrophy: natural history, MRI morphology, and dopamine receptor imaging with ^123^IBZM-SPECT. Journal of Neurology, Neurosurgery and Psychiatry.

[bib33] Spulber G., Niskanen E., Macdonald S., Kivipelto M., Padilla D.F., Julkunen V., Hallikainen M., Vanninen R., Wahlund L.O., Soininen H. (2012). Evolution of global and local grey matter atrophy on serial MRI scans during the progression from MCI to AD. Current Alzheimer Research.

[bib34] Stoodley C.J. (2012). The cerebellum and cognition: evidence from functional imaging studies. Cerebellum (London, England).

[bib35] Stoodley C.J., Valera E.M., Schmahmann J.D. (2010). An fMRI study of intra-individual functional topography in the human cerebellum. Behavioural Neurology.

[bib36] Takahashi R., Ishii K., Miyamoto N., Yoshikawa T., Shimada K., Ohkawa S., Kakigi T., Yokoyama K. (2010). Measurement of gray and white matter atrophy in dementia with Lewy bodies using diffeomorphic anatomic registration through exponentiated lie algebra: a comparison with conventional voxel-based morphometry. AJNR American Journal of Neuroradiology.

[bib37] Taylor J.P., Colloby S.J., McKeith I.G., O׳Brien J.T. (2013). Covariant perfusion patterns provide clues to the origin of cognitive fluctuations and attentional dysfunction in dementia with Lewy bodies. International Psychogeriatrics.

[bib38] Thomann P.A., Schlafer C., Seidl U., Santos V.D., Essig M., Schroder J. (2008). The cerebellum in mild cognitive impairment and Alzheimer׳s disease - a structural MRI study. Journal of Psychiatric Research.

[bib39] Tomlinson S.P., Davis N.J., Morgan H.M., Bracewell R.M. (2014). Cerebellar contributions to verbal working memory. Cerebellum (London, England).

[bib40] Walker M.P., Ayre G.A., Cummings J.L., Wesnes K., McKeith I.G., O׳Brien J.T., Ballard C.G. (2000). The clinician assessment of fluctuation and the one day fluctuation assessment scale. Two methods to assess fluctuating confusion in dementia. British Journal of Psychiatry.

[bib41] Watson R., O׳Brien J.T., Barber R., Blamire A.M. (2012). Patterns of gray matter atrophy in dementia with Lewy bodies: a voxel-based morphometry study. International Psychogeriatrics.

[bib42] Whitwell J.L., Weigand S.D., Shiung M.M., Boeve B.F., Ferman T.J., Smith G.E., Knopman D.S., Petersen R.C., Benarroch E.E., Josephs K.A., Jack C.R. (2007). Focal atrophy in dementia with Lewy bodies on MRI: a distinct pattern from Alzheimer׳s disease. Brain.

